# Early life phenobarbital exposure dysregulates the hippocampal transcriptome

**DOI:** 10.3389/fphar.2024.1340691

**Published:** 2024-03-28

**Authors:** Seán Quinlan, Tahiyana Khan, David McFall, Carolina Campos-Rodriguez, Patrick A. Forcelli

**Affiliations:** ^1^ Department of Physiology and Pharmacology, Georgetown University, Washington, DC, United States; ^2^ Interdisciplinary Program in Neuroscience, Georgetown University, Washington, DC, United States; ^3^ Department of Neuroscience, Georgetown University, Washington, DC, United States

**Keywords:** neonatal, seizure, phenobarbital, levetiracetam, toxicity

## Abstract

**Introduction:** Phenobarbital (PB) and levetiracetam (LEV) are the first-line therapies for neonates with diagnosed seizures, however, a growing body of evidence shows that these drugs given during critical developmental windows trigger lasting molecular changes in the brain. While the targets and mechanism of action of these drugs are well understood-what is not known is how these drugs alter the transcriptomic landscape, and therefore molecular profile/gene expression during these critical windows of neurodevelopment. PB is associated with a range of neurotoxic effects in developing animals, from cell death to altered synaptic development to lasting behavioral impairment. LEV does not produce these effects.

**Methods:** Here we evaluated the effects of PB and Lev on the hippocampal transcriptome by RNA sequencing. Neonatal rat pups were given a single dose of PB, Lev or vehicle and sacrificed 72 h later-at time at which drug is expected to be cleared.

**Results:** We found PB induces broad changes in the transcriptomic profile (124 differentially expressed transcripts), as compared to relatively small changes in LEV-treated animals (15 transcripts). PB exposure decreased GABAergic and oligodendrocyte markers *pvalb* and *opalin*, and increased the marker of activated microglia, *cd68* and the astrocyte- associated gene *vegfa*. These data are consistent with the existing literature showing developmental neurotoxicity associated with PB, but not LEV.

**Discussion:** The widespread change in gene expression after PB, which affected transcripts reflective of multiple cell types, may provide a link between acute drug administration and lasting drug toxicity.

## 1 Introduction

A large and growing body of evidence suggests that exposure to anti-seizure medications (ASMs) during defined and vulnerable periods of brain development induces long-lasting alterations in brain structure and function (Farwell et al., 1990; Ikonomidou et al., 2007; Meador et al., 2009; Meador et al., 2012; Meador et al., 2013; Forcelli et al., 2012a; Forcelli et al., 2012b; Al-Muhtasib et al., 2018). This is of particular concern for the treatment of neonatal seizures, which are often aggressively treated with AMSs. Phenobarbital (PB) and levetiracetam (LEV) are two of the most common therapies for neonatal seizures (Pressler et al., 2023). The most recent consensus guidance from the International League Against Epilepsy recommends PB as first line therapy, and LEV as a second-line therapy based on clinical evidence for efficacy. However, both clinical and preclinical evidence suggests that they have different safety profiles for the developing brain (Kim et al., 2007; Kaushal et al., 2016; Sharpe et al., 2020).

PB induces a profound increase in apoptosis in both developing grey and white matter when given to neonatal rats (Bittigau et al., 2002; Forcelli et al., 2011; Kaushal et al., 2016) or macaques (Ikonomidou et al., 2019; 2022). Levetericatam does not, even at doses several times those that are therapeutically relevant (Manthey et al., 2005; Kim et al., 2007). A single exposure to PB produces a disruption in synaptic development that far outlasts the period of drug exposure, reducing both excitatory and inhibitory synaptic connections in the striatum (Forcelli et al., 2012a), and hippocampus (Al-Muhtasib et al., 2018). Levetericatam does not. Both brief, and prolonged early postnatal exposure to PB produce robust and lasting deficits in learning and memory, sensorimotor gatings, and anxiety-like responses in rodents (Pick and Yanai, 1984; Pereira de Vasconcelos et al., 1990; Rogel-Fuchs et al., 1992; Frankel et al., 1995; Stefovska et al., 2008; Forcelli et al., 2012b; Bhardwaj et al., 2012; Gutherz et al., 2014). Phenobarbital also suppresses postnatal neurogenesis (Stefovska et al., 2008; Chen et al., 2009). LEV is less studied for behavioral teratogenesis, but existing evidence suggests that its profile is more benign (Manent et al., 2008; Ozyurek et al., 2010). Clinically, PB exposure in the early life period has been associated with reduced IQ (Farwell et al., 1990; Reinisch et al., 1995; Sulzbacher et al., 1999), and a metanalysis indicates better neurodevelopmental outcomes associated with LEV and compared to PB (Qiao et al., 2021).

How then, does acute drug exposure lead to long lasting changes in circuit function? One study has taken a proteomic approach and identified a set of changes in the cortical proteome immediately, 1 week, and 4 weeks following a single exposure to PB. Alterations in astrocyte markers, and synaptic proteins were observed, some lasting across time-points (Kaindl et al., 2008). A direct head-to-head comparison between a PB and LEV, has also yet to be performed. To address this gap, we turned to a transcriptomic approach. We compared the hippocampal transcriptome after a single exposure to PB, LEV, or vehicle in postnatal day (P)7 in rats. We found a robust and large set of differentially regulated genes in PB-exposed animals, and a smaller set of differentially regulated genes in LEV exposed rats 72 h after drug exposure. These data provide a link between acute drug effects, intermediate-term changes gene expression, and long term-changes in brain function.

## 2 Materials and methods

### 2.1 Drug treatments

Timed pregnant female Sprague Dawley (E15) rats were purchased from Envigo/Charles River, at postnatal day 7 (P7) male pups were weighed and randomly assigned to treatment group. Animals were purchased over two orders and one animal per litter was taken per treatment group, one for RNA sequencing and one for qPCR and immunofluorescent analysis. The P7 time point represents the period of peak vulnerability to drug-induced apoptosis (Bittigau et al., 2002) and roughly corresponds to the peak of the “brain growth spurt,” which equates to the late third trimester through early infancy in humans (Dobbing and Sands, 1979).

Treatments were balanced within litter. Animals were housed in the Division of Comparative Medicine in a temperature-controlled room (21°C) on a standard 12:12 h light-dark cycle (Lights on 0700). Dams had *ad libitium* access to food (LabDiet #5001) and water. Pups were treated with an intraperitoneal injection of LEV (200 mg/kg; 20 mg/mL), PB (75 mg/kg, 7.5 mg/mL) or vehicle (saline), and then returned to dam. LEV was purchased from Sigma-Aldrich (Product # L8668), as was phenobarbital (Product #1636). Animals were numbered using a marker on the back and tail, which was refreshed daily as needed until euthanasia (or weaning, see below).

For experiments in adult animals that received neonatal drug treatment, animals were treated as above, weaned into same-sex cages on postnatal day 21, ear tagged, and maintained in housing rooms until euthanasia at postnatal day 90.

This study was conducted under a protocol approved by the Georgetown University Animal Care and Use Committee (2016-1306) and in accordance with the Guide for the Care and Use of Laboratory Animals (National Research Council, 2011).

The dose of PB (75 mg/kg) falls at the high end of the anticonvulsant dose range in neonatal rats, induces neuronal apoptosis in developing rats, impairs both hippocampal and striatal synaptic development, and is associated with lasting behavioral changes (Kubova and Mares, 1991; Bittigau et al., 2003; Forcelli et al., 2011). Clinically, reports have found PB to be effective on a range of doses from 15 to 80 mg/kg (Kubova and Mares, 1991; Velísek et al., 1992; Polásek et al., 1996; Johne et al., 2021). On the basis of body surface area scaling the dose we used (75 mg/kg) would fall within the human 20–25 mg/kg bolus dose range (Tien et al., 2015). This dose produces plasma levels that fall within the human target range over a period of 24 h (Sanchez Brualla et al., 2023). Importantly, changes in transcriptome outlast the duration of drug action in our present study as the half-life of PB is ∼12 h.

The dose of LEV (200 mg/kg) was selected to fall at a supratherapeutic dose. This dose is expected to produce peak plasma levels in the range of 300 ug/mL, on the basis of studies in adult rats (Coles et al., 2023), which is well beyond peak levels typically observed in humans. However, LEV has a short half-life as compared to PB (e.g., ∼3 vs. 12 h). Thus, a single dose was expected to produce anti-seizure relevant effects for at least 12 h (4 half-lives). Moreover, given that prior studies have demonstrated that even supratherapeutic doses of LEV do not induce cell death or disrupt synaptic development (Manthey et al., 2005; Kim et al., 2007; Kaushal et al., 2016), this dose level provided the most robust test for a safety signal.

### 2.2 mRNA sequencing

At P10 pups were euthanized with overdose of sodium pentobarbital (Euthasol), transcardially perfused with cold PBS and hippocampi were dissected and placed in Trizol. Tissue was collected between 0900 and 1800, depending on the time of the initial treatment. Total RNA was extracted from the hippocampus using the Trizol method. RNA quantity was measured using a Nanodrop Spectrophotometer (Thermo Fisher Scientific, Waltham, MA, United States). Only samples with an absorbance ratio at 260/280 between 1.8 and 2.2 were considered acceptable. RNA degradation was assessed using bio-analyzer, all samples sent for sequencing had an RNA Integrity Number (RIN value) > 9.0.

Samples were sent to Novogene ltd. (Ca.) for sequencing. 1 mg of RNA per samples was used to generate libraries using NEBNext Ultra RNA Library Prep Kit for Illumina^®^ (NEB, United States), the library preparations were sequenced on an Illumina platform (NovaSeq 6000) and 150 bp paired-end reads were generated for an average of 24.6 M reads per sample.

Bioinformatic analyses were performed by SQ and PAF using the PartekFlow bioinformatic platform (Chesterfield, MO). Raw reads were (fastq files) were processed for QA/QC assessment (removing adaptor sequences, poly-N reads and low-quality reads) and mapped to reference rat (*rattus norvegicus*, rn6) genome using the STAR (STAR 2.7.3a) method (Dobin et al., 2013). Aligned transcripts were quantified to the rn6 annotation model using the quantify to annotation model (Partek E/M) module in PartekFlow.

For cell-type specific gene lists, we drew GABA neuron genes from (Seol et al., 2023). We added Sst, which was included in their neuron list but not in the GABA neuron list, on the basis of its strong expression in a subpopulation of interneurons. The microglial and oligodendrocyte gene lists were drawn from the same source without alteration. We used a gene list encompassing the top 50% of genes identified as most selective for astrocytes from (McKenzie et al., 2018). The meta-analysis they performed for astrocyte gene lists was much more comprehensive. These lists were not used for quantitative analysis, but rather to highlight a subset of differentially expressed genes across common CNS cell types. The final transcript lists for each cell type are shown in Supplementary Table S1.

### 2.3 qPCR

For qPCR analyses, a separate cohort of animals were treated with ASMs at P7 and sacrificed at P10- as with RNA sequencing. Total RNA was extracted with Trizol as for RNA sequencing, and cDNA synthesized using SuperScriptIV (Invitrogen) and random hexamer primers as per the manufacturers protocol. PCR reactions were prepared in duplicate using a SensiFASTTM Probe No-ROX Kit mix (Bioline, Meridian BioScience, United States) and multiplexed analyses of multiple mRNA targets within the same reaction (i.e., *actb*, *gad1* and *pvalb*) with Fluorophore containing primers from Bio-Rad, in a Mic qPCR system (Bio Molecular Systems, Australia) (Supplementary Table S5). Cycle threshold (cT) was automatically determined and averaged across replicates by the cycler manager software (Bio Molecular Sciences). Fold changes were determined using the 2^−ΔΔCT^ method, with expression of all transcripts normalized to *actb* levels in the control group.

### 2.4 Immunofluorescence

Animals were anesthetized with pentobarbital (>100 mg/kg) and transcardially perfused with cold PBS, followed by 4% paraformaldehyde (PFA, 18,505, Ted Pella Inc.). Brains were extracted and post-fixed in PFA overnight. Following post-fixation, brains were cryoprotected overnight in 15% sucrose, and then overnight 30% sucrose in PBS before flash-freezing on dry ice.

Brains were sectioned at 30 μm on a cryostat (CM 1850, Leica Biosystems) and immediately mounted on charged slides. Sections were permeabilized in PBS +0.5% TritonX100 and then blocked in a solution of PBS +2.5% normal goat serum. Sections were incubated in primary antibody overnight (Supplementary Table S6). Slices were washed and then incubated at room temperature for 1 h with secondary antibodies (Supplementary Table S6) and coverslipped with Fluoromount mounting medium containing DAPI.

Fluorescent photomicrographs were collected on a Zeiss AxioScope M2 Microscope with an Apotome optical sectioning device using Microlucida software. Images were taken at ×10 magnification (1.124 mm^2^ region). For parvalbumin positive cell counting analyses, a ×10 image was taken of the hippocampus on 3–4 non-consecutive slices (90 μm apart) and counts within CA1, CA3, and dentate gyrus were averaged per individual brain. To provide a whole-hippocampus view for presentation purposes, hippocampal sections were also scanned using a Leica Mica workstation. For CD68 and Opalin fluorescent intensity analysis, 4–6 ×10 over-lapping images were taken of the corpus collosum from the CA1 to the CA3 regions on the hippocampus on 3–4 non-consecutive slices and results were averaged per individual sample. For GFAP fluorescent intensity analysis, one 10X image was taken on the apex of the CA1 and CA3 regions and one of the dentate gyrus/hilar regions of the hippocampus (representative images of the CA3 region only) and intensity was averaged over entire hippocampal region per brain slice, with 3–4 slices per sample. Histological analysis was performed while blind to treatment status of the animal.

### 2.5 Statistics

For RNA sequencing, one sample from the control condition was excluded due to its average coverage (58.34) being double the samples (25.82–28.68), with the genomic coverage (4.939) half that of the rest of the samples (9.02–10.932). This resulted in final sample sizes for the groups of: VEH = 13, PB = 14, LEV = 14. Data were batch-normalized using a linear model function in PartekFlow to remove both main effects of batch and batch-by-treatment interactions. Differential expression analysis on the normalized data was performed using DESeq2 package (Love et al., 2014) in PartekFlow (PB v VEH; LEV v VEH). *p*-values were adjusted with the Benjamini and Hochberg approach for controlling the False Discovery Rate (FDR). Transcripts found by DESeq2 with an adjusted *p*-value <0.05 and a fold change <1.25 were considered differentially expressed.

qPCR and immunofluorescence results were analyzed by one way analysis of variance with Holm-Sidak corrected post-tests in GraphPad Prism. *p*-values <0.05 were considered statistically significant.

## 3 Results

### 3.1 Quality control and clustering

**FIGURE 1:**
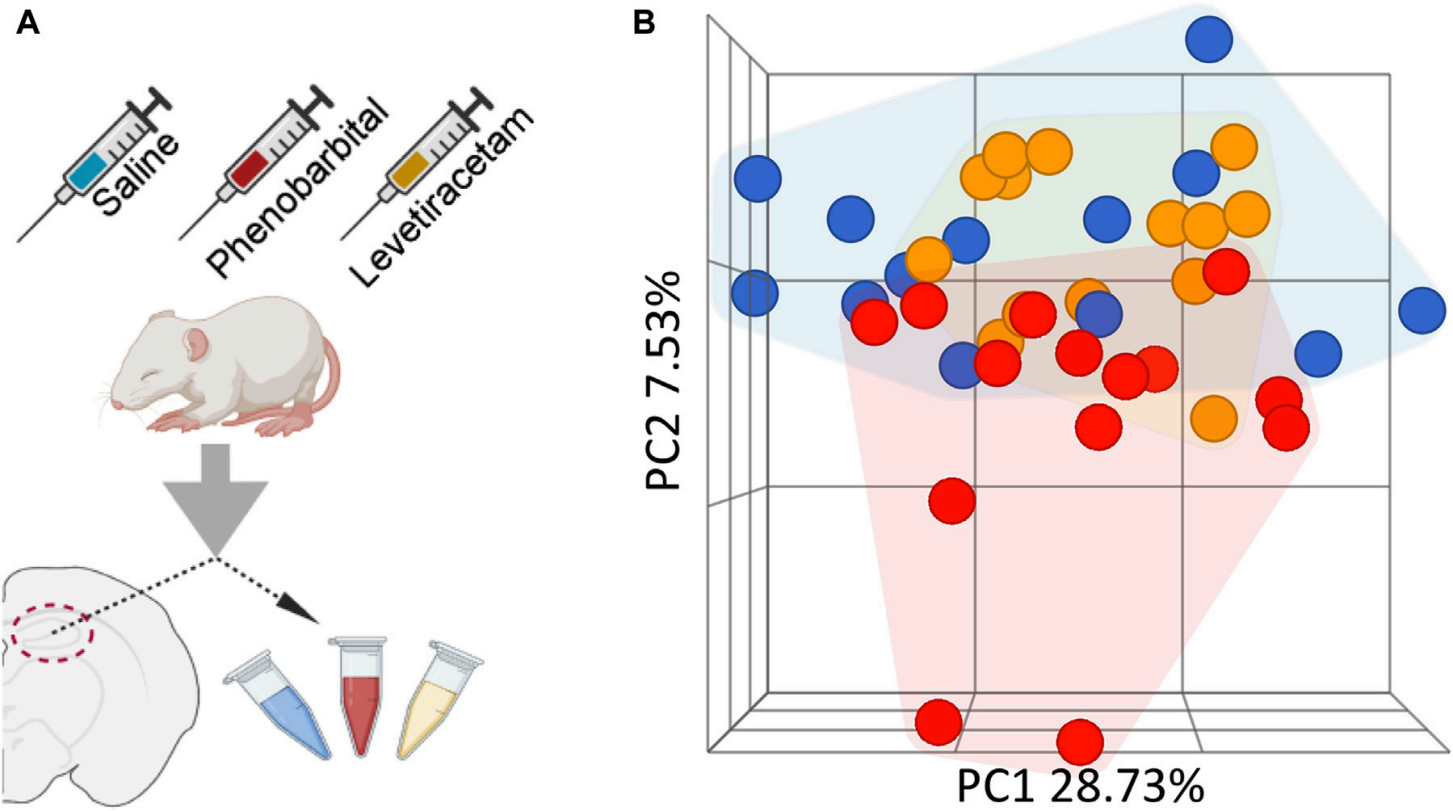
Overview of experimental design and processing. **(A)** Neonatal P7 Sprague Dawley pups treated with vehicle, PB or LEV. Schematic of P7 animals treated with drug and hippocampi harvested and RNA extracted and sequenced. **(B)** Principal component analysis of vehicle, PB and LEV treated animals.

To identify changes following anti-seizure drug administration (PB or LEV vs. vehicle control, VEH), RNA sequencing was performed on hippocampi 72 h after drug treatment (Figure 1A). Pre-alignment quality assessment and control showed a minimum of 20 million reads of 150 base pair read length transcripts per sample, with average read quality >35.0. Over 90% of aligned reads were uniquely paired. Principal component analysis (PCA) showed relatively clear clustering with the VEH and PB groups, however LEV group showed overlap with both PB and LEV groups (Figure 1B).

### 3.2 Differential gene expression following ASM exposure

**FIGURE 2:**
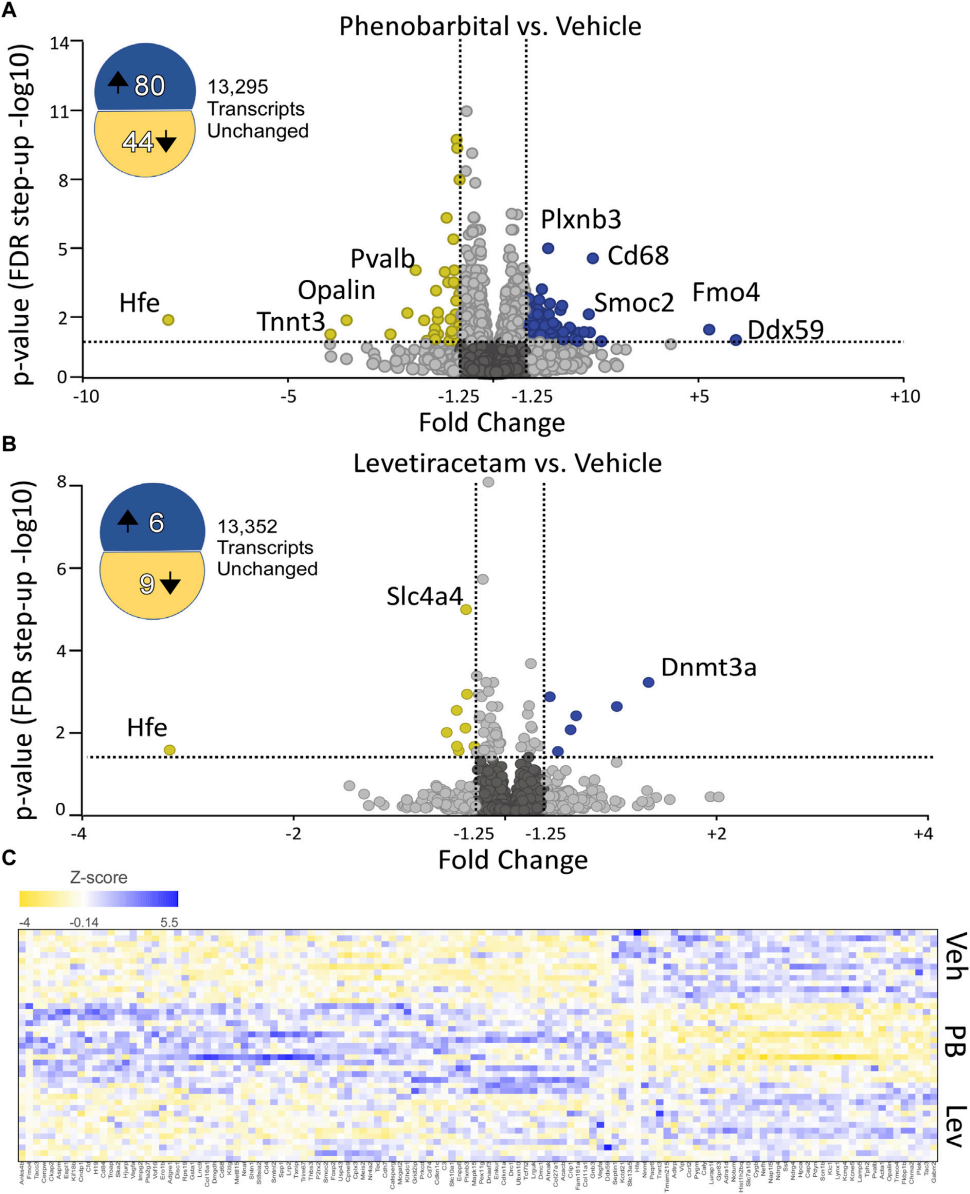
Phenobarbital robustly dysregulates the hippocampal transcriptome, while levetiracetam does not. **(A)** Volcano plot showing differential expression (PB vs. VEH). PB exposure resulted in 8- upregulated and 44 downregulated transcripts. FDR cutoff = 0.05, Fold change cutoff: -1.25 to 1.25. **(B)** Volcano plot showing differential expression (LEV vs. VEH). LEV exposure upregulated 6 transcripts and downregulated 9 transcripts. The top 10 up and downregulated genes for PHB and LEV are shown in Tables 1, 2. Full lists of transcripts are shown in Supplementary Table S2; differentially expressed transcripts following PB treatment are shown in Supplementary Table S3; differentially expressed transcripts following LEV treatment are shown in Supplementary Table S4. **(C)** Heatmap showing similar expression patterns between VEH and LEV, which differs from the pattern observed with PB.

To identify significantly differentially expressed genes (DEGs) regulated by ASM treatment, we performed differential expression analysis using the DESeq2 package. Genes with fold change ± 1.25 and an adjusted *p*-value of 0.05 were considered differentially expressed. In PB-treated animals, 124 transcripts were significantly altered as compared to controls (80 upregulated, 44 downregulated; Figure 2A). By contrast, in LEV-treated animals, we identified only 15 transcripts that were significantly altered (6 upregulated, 9 downregulated; Figure 2B). A heatmap showing the pattern of expression across groups is shown in Figure 2C. The top 10 up and downregulated genes for PB and LEV are shown in Table 1, 2. Full lists of transcripts are shown in Supplementary Table S2; differentially expressed transcripts following PB treatment are shown in Supplementary Table S3; differentially expressed transcripts following LEV treatment are shown in Supplementary Table S4.

**TABLE 1:** Top differentially expressed genes after PB exposure.

Top downregulated genes after PB exposure		
Gene symbol	P-Value	Fold change (PB vs Veh)
Hfe	1.81E-04	−8.98
Tnnt3	1.34E-03	−3.00
Opalin	1.87E-04	−2.69
Ccrl2	1.32E-03	−2.00
Pvalb	6.56E-05	−1.79
Plek	1.91E-07	−1.69
Kcng4	1.91E-04	−1.59
Paqr6	3.34E-03	−1.57
Septin1	3.92E-03	−1.54
Nnmt	1.47E-03	−1.49
Top upregulated genes after PB exposure		
Gene symbol	P-Value	Fold change (PB vs Veh)
Impg2	2.74E-03	1.73
Vegfa	3.56E-03	1.77
Cplx3	1.04E-03	1.78
Vegfa	9.33E-04	1.85
Smoc2	7.75E-05	1.91
Cfd	9.90E-04	1.92
Cd68	4.55E-08	1.96
Slc10a1	3.70E-03	2.08
Fmo4	6.97E-04	4.30
Ddx59	3.04E-03	5.15

**TABLE 2:** Top differentially expressed genes after LEV exposure.

Top downregulated genes after LEV exposure		
Gene symbol	P-Value	Fold change (PB vs Veh)
Hfe	1.42E-04	−9.31
LOC310926	3.38E-05	−1.51
Zbtb20	4.97E-06	−1.42
Rn18s	1.00E-04	−1.42
Pramef8	1.58E-04	−1.40
Slc1a2	2.15E-05	−1.34
Slc4a4	2.96E-09	−1.33
Grin2b	1.13E-06	−1.33
Slc1a2	1.04E-04	−1.26
Top upregulated genes after LEV exposure		
Gene symbol	P-Value	Fold change (PB vs Veh)
Tpcn2	1.58E-06	1.30
Fos	1.73E-04	1.37
Cpne9	2.61E-05	1.49
Npas4	7.50E-06	1.54
Pah	3.75E-06	2.02
Dnmt3a	4.55E-07	2.48
Tpcn2	1.58E-06	1.30

### 3.3 Pathway analysis

**FIGURE 3:**
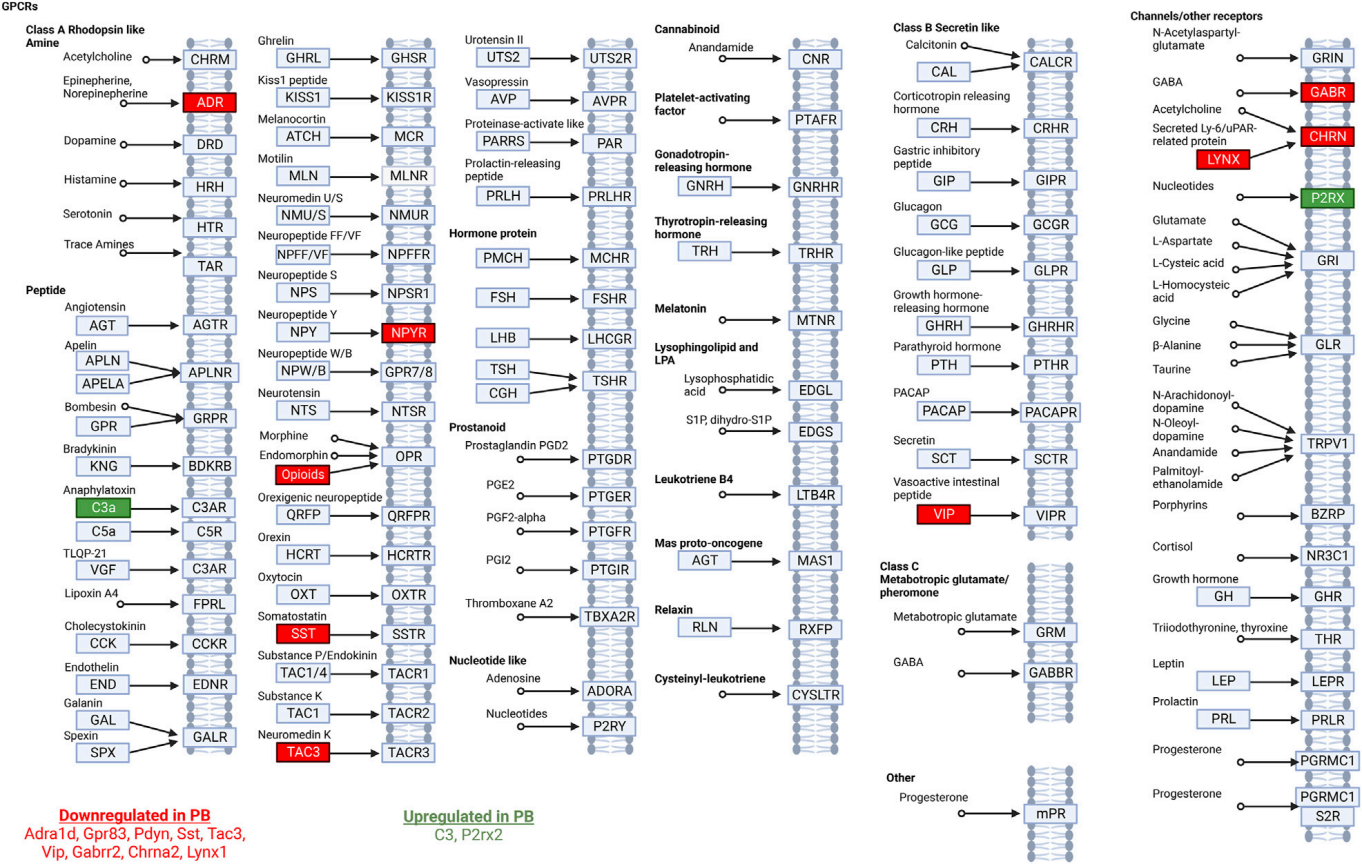
Neuroactive ligand-receptor interaction pathway analysis. Modified from the Kyoto Encyclopedia of Genes and Genomes ligand-receptor interaction pathway. Significantly DEGs in this pathway in animals treated with PB are listed at the bottom of the figure. Downregulated transcripts are shown in red and upregulated transcripts are shown in green.

We next entered the differentially expressed transcripts into a gene set enrichment analysis against the KEGG pathway database (Kanehisa and Goto, 2000; Kanehisa et al., 2023). After adjusting for multiple comparisons, the only pathway with significant enrichment was the neuroactive ligand-receptor interaction pathway (rno04080) with an enrichment score of 9.3 (*adj. p-value =* 0.03) for differentially expressed genes in the PB treated group (Figure 3). 3.6% (11 of 305) of genes in the set were differentially expressed following PB treatment.

### 3.4 GABAergic neuron associated transcripts are downregulated following PB treatment

**FIGURE 4:**
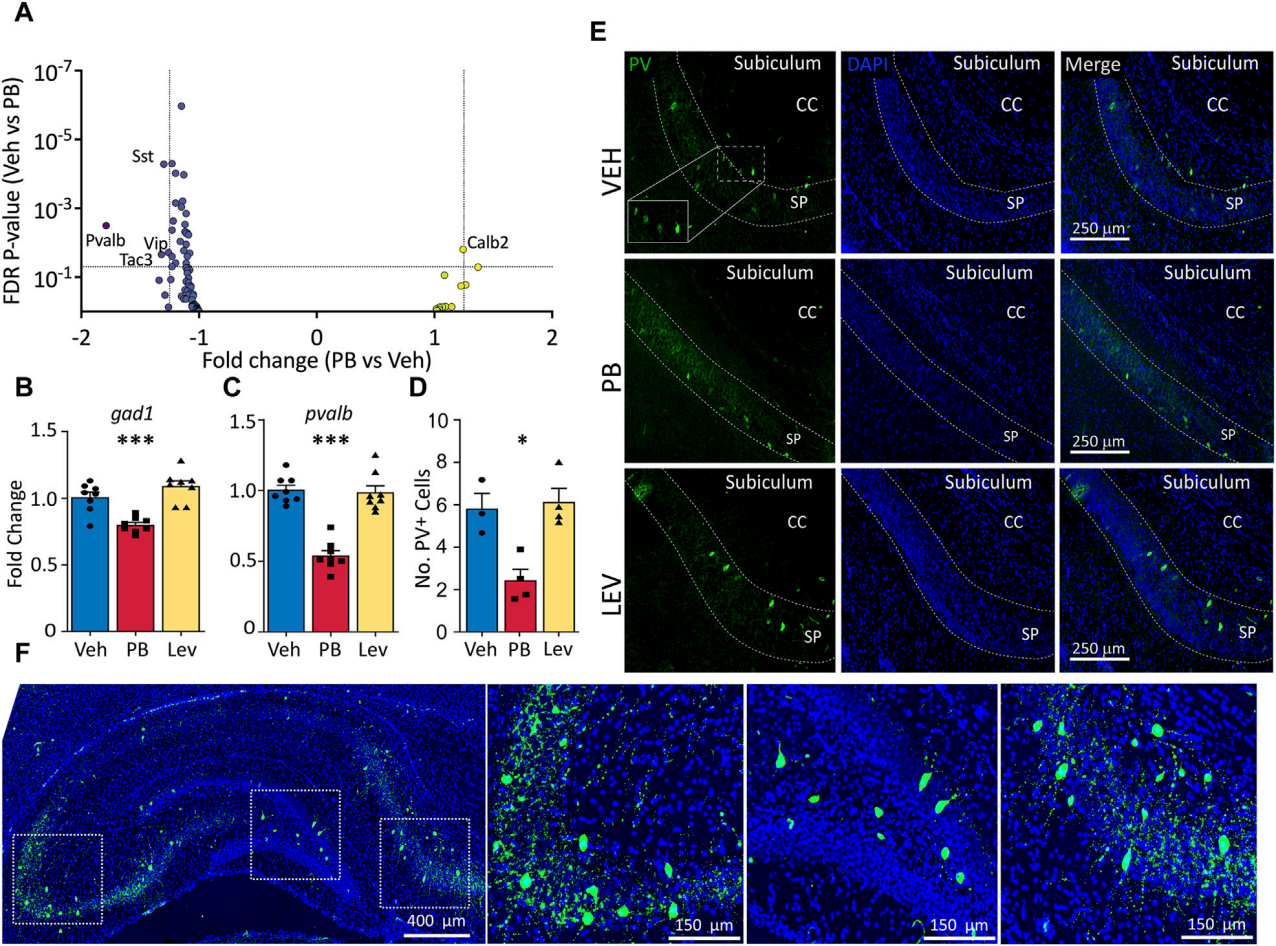
Specific decrease in pvalb GABAergic transcripts in PB, but not Lev treated animals. **(A)** Animals treated with PB, but not with LEV showed significant dysregulation of GABAergic neuron related genes. **(B,C)** qPCR analysis confirmed that PB treatment significantly reduced both general GABAergic marker *gad1* transcripts (One Way ANOVA, F_2,21_ = 18.97, *p*-value <0.0001, VEH vs. PB adj. *p*-value = 0.0007 with Holm-Sidak corrected) and reduction of *pvalb* transcripts (One Way ANOVA, F_2,21_ = 43.34, *p*-value <0.0001, VEH vs. PB adj. *p*-value <0.0001, Holm-Sidak corrected). **(D,E)** Representative photomicrographs of parvalbumin (left column) and DAPI (middle column) staining in the hippocampus in animals treated with vehicle (top row), PB (middle row) and LEV (bottom row). Animals treated with PB showed significantly lower levels of immunoreactivity compared to vehicle treated animals (One Way ANOVA, F_2,8_ = 11.3, *p*-value <0.0047, adj. *p*-value = 0.012, Holm-Sidak corrected). CC = corpus callosum, SP = stratum pyramidale of CA1. **(F)** Representative image showing the pattern of PV expression in a whole hippocampus section with enlarged views of subfields.

We had previously reported altered GABAergic synaptic development in the hippocampus after exposure to PB (Al-Muhtasib et al., 2018). Given this prior finding, and the presence of GABA-neuron related genes in our KEGG analysis (above), we next sought to evaluate the impact of PB and LEV on GABAergic markers. Figure 4A shows a volcano plot with genes from a GABA neuron gene list for the PB condition (Supplementary Table S1). Four of 88 genes were downregulated in PB-exposed animals (Sst, Pvalb, Vip, Tac3). Calb2 fell just under the fold-change cutoff (1.24) for increased expression in PB-exposed animals. No transcripts were altered in the LEV-exposed condition.

To verify our RNA-seq results we selected general markers of GABAergic cells (*gad1*) and specific markers of parvalbumin expressing GABA cells (*pvalb*) to evaluated by qRT-PCR (Figures 4B, C). *gad1* mRNA levels differed across groups (One Way ANOVA, F_2,21_ = 18.97, *p*-value <0.0001), an effect that was driven by a significant decrease in the PB-treated group (*p*-value = 0.0007, Holm-Sidak corrected). *gad1* mRNA levels did not differ between LEV and VEH treated animals (*p*-value = 0.103, Holm-Sidak corrected). We observed a similar pattern with *pvalb* mRNA, with the difference among the groups (One Way ANOVA, F_2,21_ = 43.34, *p*-value <0.0001), driven by a downregulation in the PB treated animals (*p*-value <0.0001, Holm-Sidak corrected).

To determine if PB causes a reduction in *pvalb* transcripts only, or if this decrease was also associated with changes at the protein level, we performed immunofluorescence of parvalbumin and analyzed the number of expressing cells in the hippocampus (average of CA1, CA3, DG; Figures 4D, E). Similar to our PCR results, we found a significant difference amongst the groups (One Way ANOVA, F_2,8_ = 11.3, *p*-value <0.0047), which was driven by a significant decrease in the number of PV^+^ cells compared to the VEH group (*p*-value = 0.012, Holm-Sidak corrected). LEV treated animals did not differ from VEH treated controls (*p*-value = 0.736, Holm-Sidak corrected).

### 3.5 Microglial-enriched genes

**FIGURE 5:**
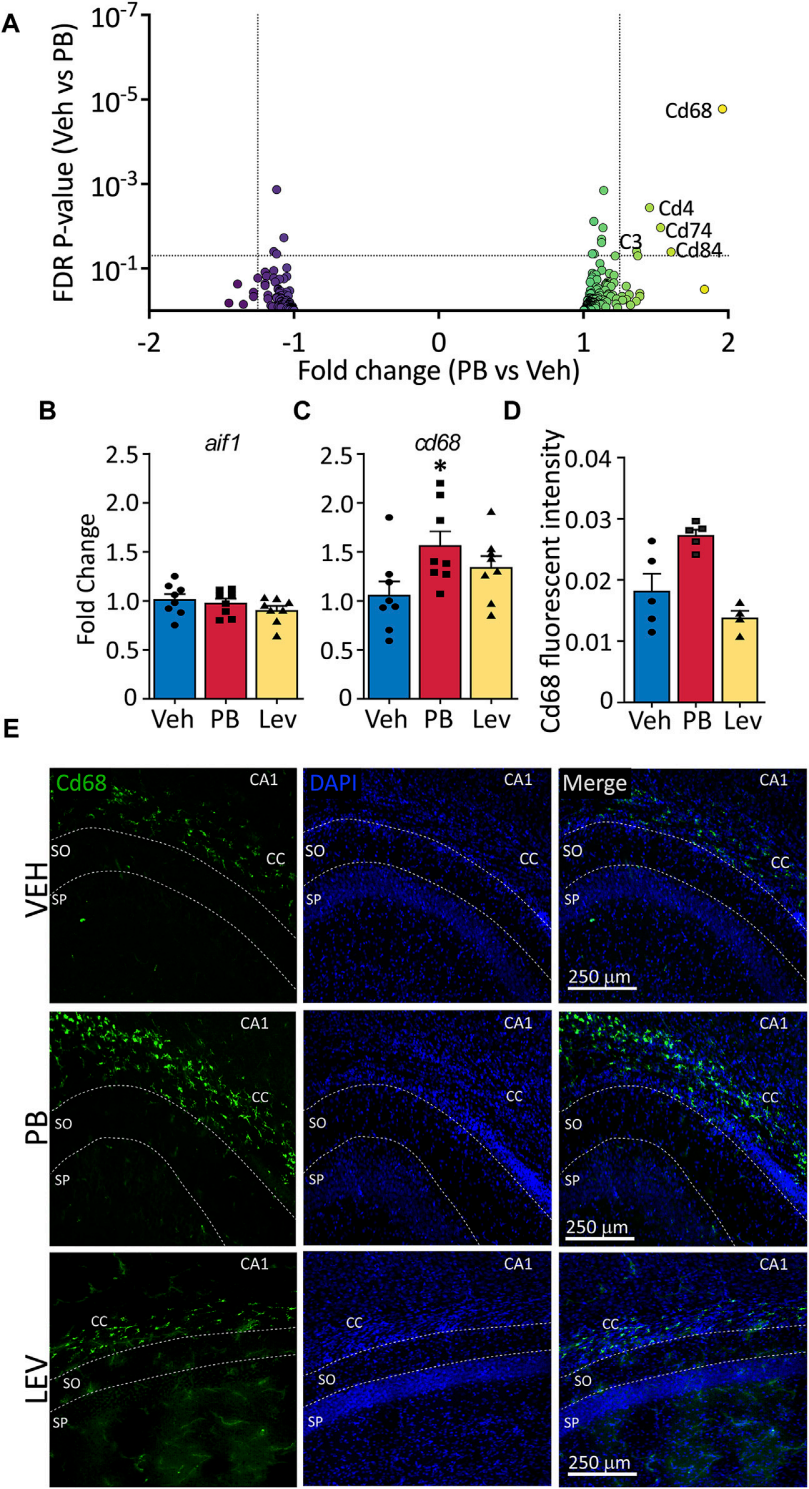
The activated microglia marker cd68 is increased following ASM treatment **(A)** Volcano plots of microglial associated genes. PB treated animals showed increased levels of the activated microglial marker, CD68, however no significant changes in any microglial markers were observed in the LEV treated group. **(B)** The general microglial marker *aif1* (iba1) was unchanged in both PB and LEV animals when analysed by qPCR. **(C)** *cd68* transcripts were increased in PB but not LEV treated animals (One Way ANOVA *p*-value = 0.048, F_(2, 21)_ = 3.53. VEH vs. PB adj. *p*-value = 0.029). **(D,E)** Representative photomicrographs of CD68 (left column) and DAPI (middle column) staining in the corpus collosum in animals treated with vehicle (top row), PB (middle row) and LEV (bottom row). Animals treated with PB showed increased CD68 immunofluorescent intensity in the CC when compared with VEH (One Way ANOVA *p*-value = 0.0015, F_(2, 11)_ = 12.36, VEH vs. PB adj. *p*-value = 0.016), while there was no difference between LEV and VEH animals. SP = stratum pyramidale of CA1, SO = stratum oriens of CA1. CC = corpus callosum.

Given that CD68, a membrane protein expressed in *activated* microglia, was upregulated in following PB treatment, and PB treatment robustly triggers cell death, which in turn may activate microglia, we further analyzed the effects of ASMs on microglial enriched genes. We examined a curated list of 267 genes enriched in microglia (Supplementary Table S1), and found that five transcripts that were differentially regulated (*Cd68, Cd4, Cd74, C3, and Cd84*). *Cd68* displayed the largest fold change from this list (+1.97, adj. *p*-value <0.0001) following PB exposure (Figure 5A). No transcripts from the list were differentially expressed in LEV exposed animals.

To confirm RNA-seq results we performed qRT-PCR on general microglial marker *Aif1* (Iba1) (Figure 5B) and activated microglial marker *Cd68* (Figure 5C). We found no difference in *Aif1* transcript levels in either PB or LEV animals as compared to VEH treated animals (One Way ANOVA, F_2,21_ = 1.287, *p*-value = 0.297). However, we found significant differences between groups for *Cd68* transcript levels (One Way ANOVA, F_2,21_ = 3.53, *p*-value = 0.0476). This difference was driven by a significant increase in the PB group when compared to the VEH group (*p*-value = 0.0296, Holm-Sidak corrected). *cd68* levels in LEV treated animals did not differ from those in VEH treated animals (*p*-value = 0.153).

We performed immunofluorescence for CD68 and found a significant difference in hippocampal CD68 fluorescence intensity between groups (One Way ANOVA, F_2,11_ = 12.36, *p*-value = 0.0015), an effect that was driven by a significant increase in the PB exposed group (*p*-value = 0.016, Holm-Sidak corrected; Figures 5D, E).

### 3.6 Astrocyte-enriched genes

**FIGURE 6:**
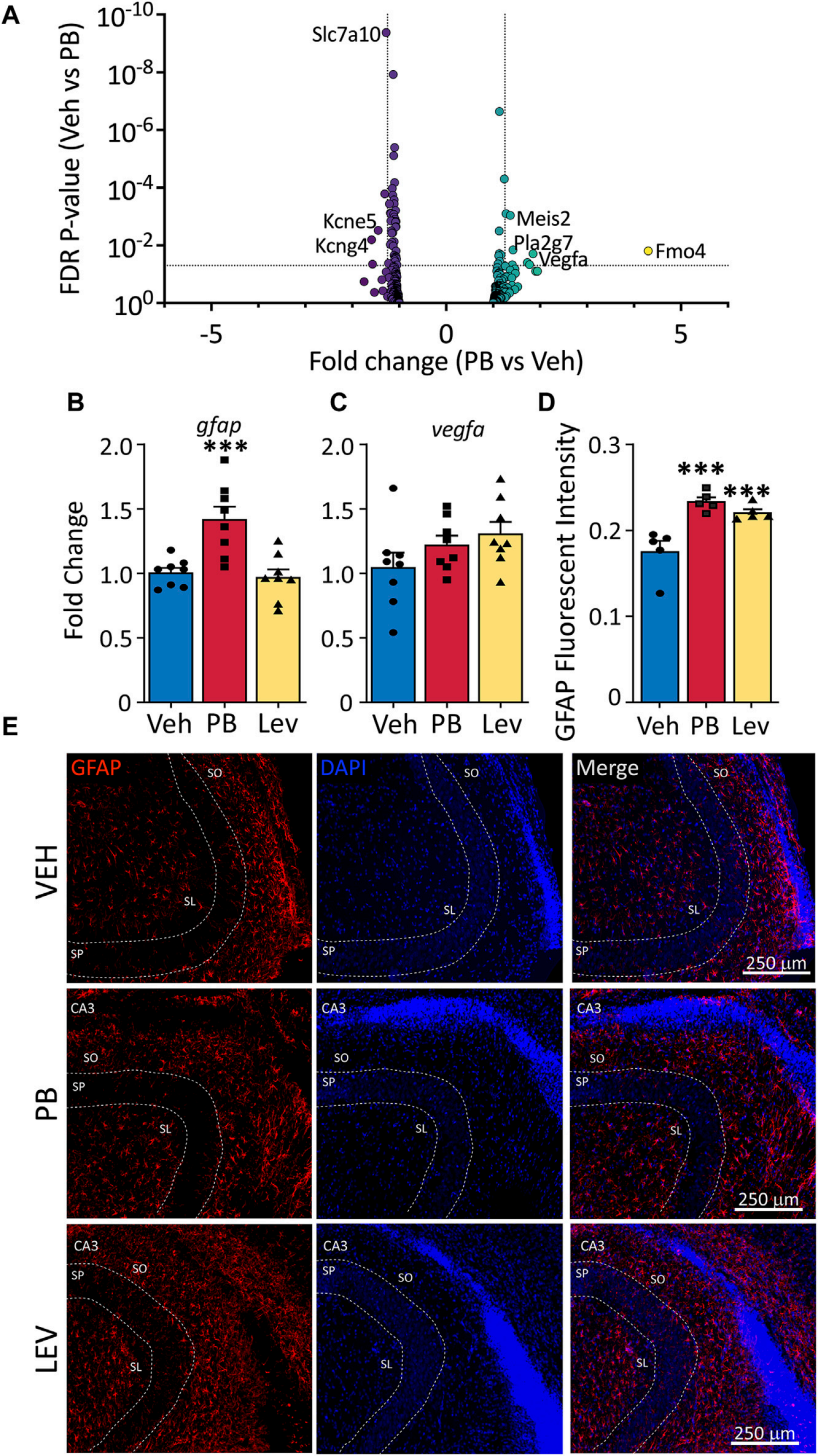
Altered astrocytic marker expression following ASM treatment. **(A)** Volcano plots of astrocyte associated genes. Transcripts of *vegfa* were found increased in both PB and LEV treated animals. **(B)** qPCR for *gfap* showed that it was increased in PB treated (One Way ANOVA *p*-value = 0.0003, F_2, 21_ = 12.15, adj. *p*-value = 0.001) but not in LEV treated animals. While *vegfa* **(C)** mRNA levels were unchanged in both treatment groups (One Way ANOVA *p*-value = 0.163, F_2, 21_ = 1.978). **(D,E)** Representative photomicrographs of GFAP (left column) and DAPI (middle column) staining in the CA3 in animals treated with vehicle (top row), PB (middle row) and LEV (bottom row). Fluorescent intensity analysis showed that animals treated with both PB and LEV have increased levels of GFAP^+^ cells when compared with vehicle treated animals (One Way ANOVA *p*-value = 0.0007, F_2, 12_ = 14.19, VEH vs. PB adj. *p*-value<0.001 and VEH vs. LEV adj. *p*-value<0.005). SP = stratum pyramidale, SO = stratum oriens, SL = stratum lacunosum-moleculare.

Astrocytes are a major cell glial type in the CNS; while astrocytes have not been directly linked to developmental toxicity of anti-seizure medications, they play a critical role in regulating synaptic transmission as part of the tripartite synapse, and the developmental trajectory of glutamatergic neurotransmission is altered following even a single exposure to PB. We evaluated a gene list enriched in astrocytes (Supplementary Table S1) and found 13 of 441 were differentially expressed following PB treatment (Figure 6A). Eight transcripts were increased following PB: two *Vegfa* transcripts (variant 7: NM_001287113; variant 9 (non-coding): NR_105011), as well as *Fmo4, Impg2, Pla2g7, Lrrc9, Meis2* and *Col16a1.* Six transcripts were decreased in expression afer PB exposure: *Slc7a10, Slc13a5, Kcne5, Paqr6, and Kcng4.* In the LEV-exposed group, four transcripts (*slc4a4* - NM_053424 and *slc1a2* - NM_001302089 and NM_001035233; and *Zbtb20* - NM_001105880) were downregulated relative to the VEH exposed group, since so few transcripts were differentially regulated with LEV, no volcano plot is shown for that condition.

We assessed *gfap* transcript levels by qPCR (One Way ANOVA, F_2,21_ = 12.15, *p*-value = 0.0003) and found group differences that were driven by a significant increase in the PB exposed animals (Figure 6B). While in our DESeq analysis, this transcript fell just below the cutoff for fold change (1.24) the *p*-value was highly significant (*p*-value = 5 × 10^−5^), consistent with our qPCR results (*p*-value = 0.0011, Holm-Sidak corrected). LEV did not differ from VEH for *gfap* transcript levels (*p*-value = 0.72).

We found no differences in drug treatments overall on the levels of *vegfa* (One Way ANOVA, F_2,21_ = 1.98, *p*-value = 0.163; Figure 6C), however this could be due to different transcript variants targeted by PCR amplification (PCR amplicons; NM_001110333, NM_031836, NM_001110334, NM_001110335 and NM_001110336). To identify if altered *Gfap* transcript levels observed in our DESeq analysis resulted in dysregulated GFAP expression in cells, we performed immunofluorescence staining for GFAP in the hippocampus (Figures 6D, E). We used relative fluorescent intensity as a measure of GFAP expression across three regions; the CA1, CA3 and dentate gyrus/hilar regions of the hippocampus, and found an overall effect of drug treatment on GFAP expression by one-way ANOVA (F_2, 12_ = 14.19, *p*-value = 0.0007). Both PB and LEV treated animals had increased fluorescent intensity of GFAP when compared to VEH animals (*p*-value’s = 0.0005 and 0.0019, Holm-Sidak adjusted, respectively).

### 3.7 Oligodendrocyte-enriched genes

**FIGURE 7:**
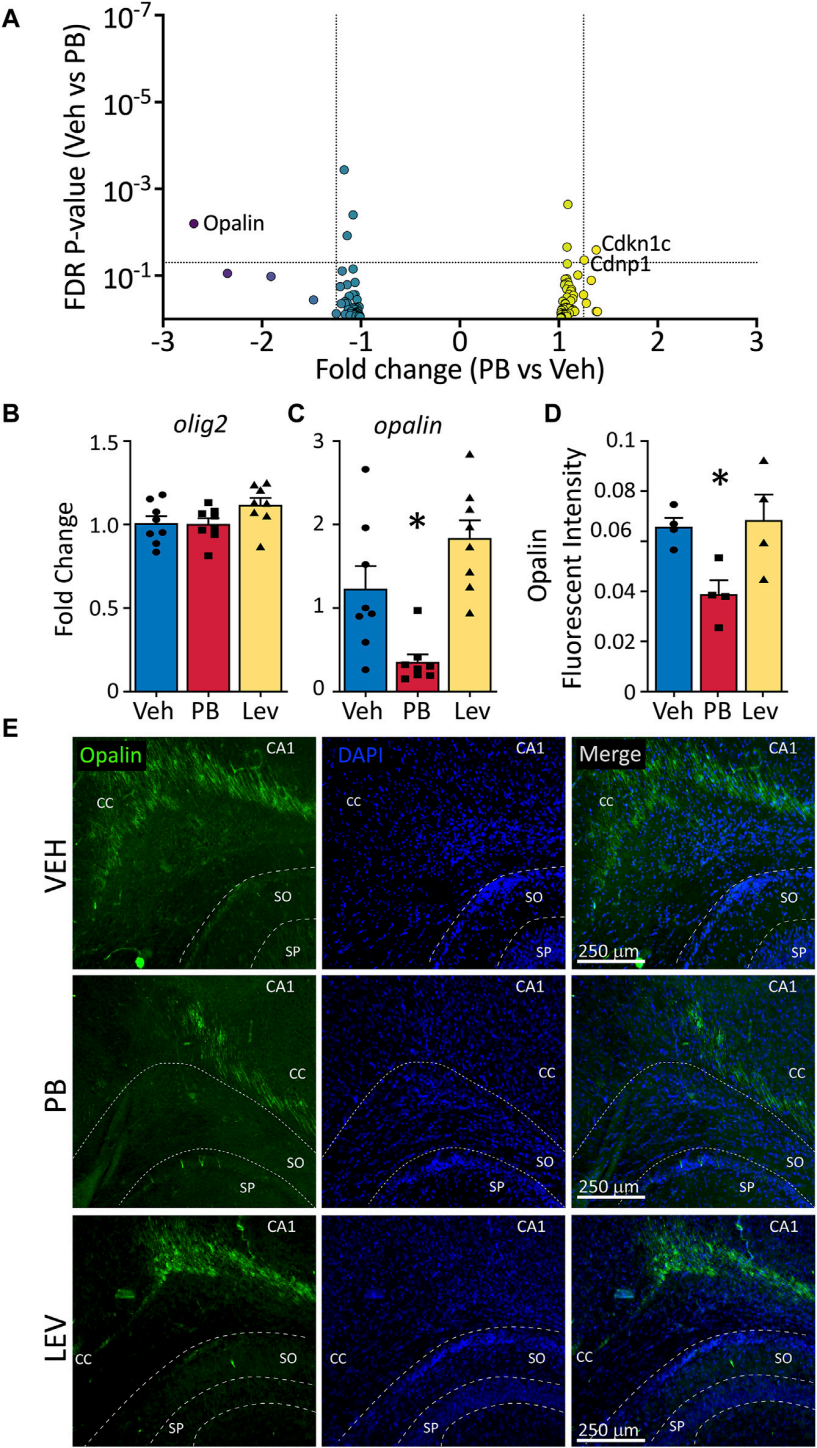
Decreased opalin expression in PB treated animals. **(A)** Volcano plots of oligodendrocyte associated genes. **(B)** The mRNA levels of *olig2* were unchanged in both PV and LEV treated animals when analyzed by qPCR (One Way ANOVA *p*-value = 0.4, F_2, 33_ = 0.94), however we observed a significant decrease in the levels of **(C)** *opalin* in PB treated animals when compared to vehicle (One Way ANOVA *p*-value = 0.0003, F_2,21_ = 12.51. adj. *p*-value = 0.02) **(D,E)** Representative photomicrographs of opalin (left column) and DAPI (middle column) staining in the corpus collosum in animals treated with vehicle (top row), PB (middle row) and LEV (bottom row). Reduced levels of opalin^+^ fluorescent intensity in the CC of animals treated with PB when compared to vehicle (One Way ANOVA *p*-value = 0.0106, F_2, 10_ = 7.416, VEH vs. PB adj. *p*-value = 0.02 Holm-Sidak corrected). SP = stratum pyramidale of CA1, SO = stratum oriens of CA1. CC = corpus callosum.

In addition to induction of neuronal apoptosis following PB exposure in neonatal animals (Bittigau et al., 2002), profound oligodendrocyte apoptosis has also been observed in developing white matter (Brambrink et al., 2012; Creeley et al., 2013; Creeley et al., 2013; Creeley et al., 2014; Ikonomidou et al., 2019). Interestingly, opalin (also referred to as TMEM10), an oligodendrocyte transmembrane protein, was the transcript with the fifth largest absolute fold change following PB exposure (Fold Change: -2.69; *p*-value <0.001). Opalin is enriched in myelin and its expression is upregulated during oligodendrocyte differentiation. We evaluated, as with neuronal and microglial gene lists, a gene list for oligodendrocytes (Supplementary Table S1) in animals treated with PB as compared to VEH (Figure 7A). Of these transcripts, three were differentially expressed: *Opalin*, *Cdkn1c, and Cndp1*. No transcripts from the list were differentially expressed in LEV exposed animals, so no volcano plot is shown.

We assessed levels of *olig2* by qPCR and found no differences across treatment groups (One Way ANOVA, F_2,33_ = 0.94, *p*-value = 0.40; Figure 7B); by contrast *opalin* differed significantly (One Way ANOVA, F_2,21_ = 12.51, *p*-value = 0.0003; Figure 7C). This effect was driven by a significant decrease in *opalin* transcript levels in the PB exposed group (adj. *p*-value = 0.0146, Holm-Sidak corrected). The LEV group did not differ from the VEH exposed group, although there was a non-significant trend toward increase *opalin* levels in the LEV condition (adj. *p*-value = 0.055). When we assessed Opalin fluorescent intensity, we again found a difference between groups (One Way ANOVA, F_2,10_ = 7.41, *p*-value = 0.0106; Figures 7D, E), which was driven by a decrease in intensity in the PB exposed group (adj-p-value = 0.02, Holm-Sidak corrected) as compared to the VEH group. LEV exposure did not alter opalin immunofluorescence (adj. *p*-value = 0.081).

### 3.8 Longer-term gene expression profiles

For the transcripts we found to be dysregulated by qPCR in the above experiments, we separately assessed their relative abundance in PB- or LEV-exposed animals 3 months after exposure. *Gfap, opalin and cd68* did not differ across treatment groups at 3 months post exposure. To our surprise, and differing from what we observed 3 days after exposure, *pvalb* was *increased* in the LEV-treated group compared to vehicle or PB exposure, and the PB-treated group did not differ from controls. *Gad1* transcript levels remained lower in the PB-treated group 3 months after drug exposure, consistent with the pattern observed 3 days after exposure (see Supplementary Figure S1 for results and statistics).

## 4 Discussion

The choice of anti-seizure medication for use in pregnancy and infancy is complicated by concerns of long-term effects on the developing brain. However, the mechanisms linking acute drug exposure to lasting changes in synaptic function, behavior, and cognitive outcome remain obscure. Here, we examined the two commonly used ASMs for the treatment of neonatal seizures, PB and LEV for effects on the hippocampal transcriptome. PB has well-established neurodevelopmental effects, whereas LEV is commonly thought to be safer. Consistent with this general notion, we found a robust change in the hippocampal transcriptome 72 h after a single exposure to PB: 124 transcripts were differentially expressed in PB-exposed animals compared to vehicle controls. By contrast, only 15 transcripts were differentially expressed in LEV-exposed animals. We followed this analysis with profiling of GABAergic neurons, microglia, astrocytes and oligodendrocytes, and for each cell type found markers that were differentially impacted at the transcript and/or protein level following PB exposure.

The changes we found outlasted the acute drug exposure (P7)—for PB ∼6 half-lives passed between treatment and tissue collection (Dingemanse et al., 1989), a point at which only ∼1% of the drug is expected to remain. These changes thus may provide a link between acute drug administration and disrupted synaptic development that we have previously reported following PB exposure. For example, P7 exposure to PB disrupts the maturation of inhibitory and excitatory synapses in the striatum and inhibitory synaptic development in hippocampus. In both regions, these effects are evident well after drug is eliminated. In striatum, this effect was manifest by a decrease in inhibitory postsynaptic current (IPSC) frequency (Forcelli et al., 2012a), whereas in the hippocampus we found an initial increase in IPSC frequency, followed by a reduced IPSC frequency at later ages indicating impaired maturation (Al-Muhtasib et al., 2018). In hippocampus, we also observed an increase in tonic GABA currents, and a long-lasting perseverance of giant depolarizing potentials, which are large GABAergic events that are normally absent by the second postnatal week (Al-Muhtasib et al., 2018). Along these lines, it is noteworthy that we identified altered expression of somatostatin, vasoactive intestinal peptide, and parvalbumin transcripts, each of which label populations of GABAergic interneurons in the hippocampus (Pelkey et al., 2017). Moreover, we found that Gpr83, a g-protein coupled receptor that binds the neuropeptide PEN (and may bind neuropeptide Y, as well), which is enriched in parvalbumin interneurons in the amygdala (Lueptow et al., 2018; Fakira et al., 2021). While PEN expression has been reported in the hippocampus, it is unknown what cell types it co-localizes with. Together, these changes suggest that PB impacts inhibitory neurotransmission, and raise the possibility that these changes could in turn, impact PB efficacy–reduced parvalbumin neurons, for example, would be expected to worsen epilepsy outcomes. Suggesting that not only inhibitory transmission may be altered, but also function of excitatory cells, we noted a change in transcript levels for pro-dynorphin, which is enriched in dentate gyrus granule cells in the hippocampus (Chavkin et al., 1985).

Programmed cell death, which is a natural part of postnatal brain development, eliminates neurons that do not establish appropriate synaptic connections. Drugs, such as PB, exacerbate this process, significantly increasing the number of degenerating cells. Microglia play a critical role in the phagocytosis of neurons that undergo programmed cell death (Ferrer et al., 1990; Bessis et al., 2007; Schafer and Stevens, 2015), in fact developmental cell death in the hippocampus requires functional microglial (Wakselman et al., 2008). Neuronal apoptosis drives dynamic changes in microglial states in the retina–and prevention of cell death interestingly *downregulates* cd68 (Anderson et al., 2022). We observed the opposite pattern with PB exposure–CD68 was upregulated at both the transcript and protein level. This effect was not observed in LEV exposed animals, consistent with the induction of cell death by PB, but not by LEV. The impact of prolonged elevation of CD68 beyond the acute injury (the peak detection of degenerating cells after PB exposure is ∼24 h), remains unstudied, but given the role for CD68-positive microglia in synaptic pruning (Schafer et al., 2012), raises the possibility that microglial activation may contribute to long-term circuit changes after PB exposure. Assessment of microglial morphology and “activation” state following PB exposure may thus be of future interest.

Interestingly, we observed increased GFAP immunoreactivity–indicative of astrocytosis–in both PB and LEV treated animals, although at the transcript level GFAP was increased only after PB exposure. The functional significance of this increased astrocyte activation is unknown–astrocyte activation is associated with both acute damage responses and repair/plasticity responses to injury (Yang and Wang, 2015). Moreover, why Lev treated animals have increased reactivity to GFAP immunofluorescence, with no detected changes in gfap transcripts remains unclear. We identified an increase in complement C3 after PB exposure (1.37 FC; *p*-value = 0.04), and astrocytes are a major source of C3 in the brain (Pekna and Pekny, 2021), furthermore C3 may be an important component of astrocyte-microglial cross signaling during states of brain damage. While it is uncertain if the elevated C3 transcript levels we detected is astrocytic in origin, it is worth noting that astrocyte C3 plays an important role in dendritic spine pruning (Lian et al., 2015) and has both putative protective and neurotoxic roles (Pekna and Pekny, 2021). Along these lines, while astrocytes do not appear to undergo programmed cell death, or display increased activation after exposure to isoflurane, an anesthetic agent, this has not been evaluated for anti-seizure medications (Brambrink et al., 2012). However, in astrocyte-neuron co-culture experiments, astrocytes were proposed to play a *critical* role in apoptosis triggered by inhibition of neuronal activity (Shute et al., 2005). In culture, as *in vivo*, ethanol, sodium channel blockers, and NMDA receptor antagonists trigger apoptosis in hippocampal neurons; reducing astrocytes in the cultures protected against this neuronal cell death, and treating cultures with astrocyte conditioned media increased cell death. While the authors of this study determined that there was a heat-labile component of astrocyte conditioned medium that was responsible for this effect, the identity of this signal remains unknown (Shute et al., 2005). Finally, the increase in Vegf transcripts we detected in our DESeq analysis suggests that blood-brain-barrier and microvascular function merits further investigation after early-life exposure to PB.

In addition to neuronal apoptosis, neonatal drug exposure (including to PB) is associated with increased apoptosis in the developing white matter (Kaushal et al., 2016), predominantly due to apoptosis of immature oligodendrocytes (Brambrink et al., 2012; Ikonomidou et al., 2022). The apoptosis (following anesthesia) preferentially impacts immature oligodendrocytes (O4-positive) (Brambrink et al., 2012); and O4-positive immature oligodendrocytes express opalin at high levels. During periods of brain growth, immature oligodendrocytes require electrical activity of axons to regulate proliferation/oligodendrogenesis (Barres and Raff, 1993), myelination (Gibson et al., 2014), oligodendrocyte precursor survival (Hill et al., 2014), and axon selection (Hines et al., 2015). Similar findings suggest that synaptic vesicle release from neurons is necessary for normal myelination (Mensch et al., 2015). While speculative, it is thus possible that disruptions in the oligodendrocyte linage (consistent with decreased opalin expression), may be a secondary consequence of neuronal inhibition by anti-seizure medications. The degree to which myelination recovers, and the long-term consequences of impaired early myelination after anti-seizure medication exposure remain to be examined.

The only other omics-based study to assess drug toxicity after early-life exposure to PB used a proteomic approach (Kaindl et al., 2008) and identified 45 peptides that were acutely or chronically altered by PB exposure on P6. Of the 45 peptides, two overlap with the transcripts we identified here: parvalbumin, which was identified in our transcriptomic analysis, and GFAP, which was identified in our qPCR/histological analysis. The prior study identified a range of transcripts that were involved in oxidative stress and apoptosis, cell cycle function, and neurite outgrowth. While we did not identify any of the same hits in our transcriptomic analysis, we did note an upregulation of the Foxp2 transcription factor by PB exposure. This transcription factor is strongly associated with speech and language disorders in humans, and it coordinates a gene network that modulates neurite outgrowth (Vernes et al., 2011). Similarly, we found increased transcript levels for Disc1, a gene associated with schizophrenia after PB exposure. Disc1 is critical for neuronal differentiation, migration, and axon/dendrite targeting and growth (Soares et al., 2011). Interestingly, some phenotypes associated with early-life PB exposure are shared with schizophrenia, including impaired sensorimotor gating (Forcelli et al., 2012b; Gutherz et al., 2014), and we have previously reported additive toxicity of PB in the neonatal ventral hippocampal lesion model of schizophrenia (Bhardwaj et al., 2012). Finally, we also noted an increase in Cdkn1a (p21), a cell cycle inhibitor which has been associated with senescence/quiescent cell fates (Jurk et al., 2012; Gillispie et al., 2021), which may impact cell recovery after drug exposure induced injury.

There are several caveats to our present study. First, because our transcriptome-wide assessment was performed 72 h after drug exposure, it remains unclear which, if any of these changes are persistent beyond this period. Of the 5 transcripts we examined 3 months after exposure, *gad1* displayed the same profile as early after exposure (decrease with PB). *Pvalb* was increased in the LEV-treated group at 3 months, but not 3 days, and the decrease observed in the PB treated group at 3 days was absent at 3 months. The other three transcripts (*cd68, opalin, gfap*) each of which were dysregulated by PB at 3 days, did not differ between groups at 3 months. That said, even brief disruptions to developmental processes may set off long-lasting alterations. Moreover, we only evaluated transcript levels at 3 months, not protein expression, and these may be decoupled. Furthermore, we examined only a small set of transcripts at 3 months. Future studies examining later timepoints are therefore warranted. Second, while our focus was on the developing brain, it is unclear which, if any of these transcriptomic changes would also be observed in adult animals treated with AMSs. However, the functional significance of these changes may also differ dramatically between the neonatal and adult brain, as again, brief disruptions to function during development can alter the trajectory of brain development. Third, the present study was conducted in seizure-naïve animals; in a clinical setting these drugs are unlikely to be given to neonates in the absence of seizures or other pathology. However, these findings are also likely relevant to exposure *in utero* as the postnatal day 7 rat models a time range approximately equivalent to the end of the third trimester of pregnancy through early infancy in humans. The degree to which drug exposure interacts with a history of seizures is of clear relevance and is the topic of another ongoing study in our laboratory.

Here we present a transcriptomic profile comparing the effects of early life exposure to the two most common anti-seizure medications used to treat neonatal seizures. PB, is associated with acute neurotoxicity, impaired synaptic development, and long-term behavioral changes, induced changes in gene expression in 124 transcripts; LEV, avoids these toxicities in the developing brain and showed changes in only 15 transcripts. The transcript domains impacted centered around neuroactive receptor-ligand interactions, and transcripts linked to neurons, microglia, oligodendrocytes, and astrocytes. Together these data may provide a link between acute early life toxicity and lasting changes in brain function after exposure to PB.

## Data Availability

The datasets presented in this study can be found in online repositories. The names of the repository/repositories and accession number(s) can be found below: https://www.ncbi.nlm.nih.gov/geo/query/acc.cgi?acc=GSE247577.
