# Supplementary material for: Early life phenobarbital exposure dysregulates the hippocampal transcriptome
Source: Front Pharmacol. 2024 Mar 28;15:1340691. doi: 10.3389/fphar.2024.1340691 (PMC11007044; doi:10.3389/fphar.2024.1340691)
Supplement: Supplementary file 7 [file Image1.pdf]

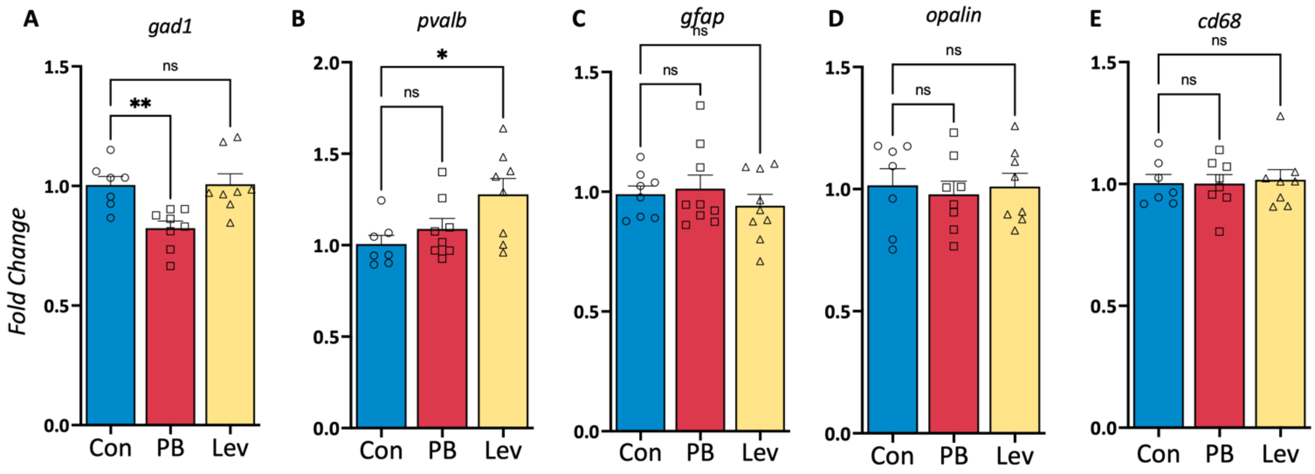

Supplementary Figure 1. Selected transcript levels measured 3 months after drug exposure. For the transcripts we found to be dysregulated by qPCR 3 days after drug exposure, we separately assessed their relative abundance in PB- or LEV-exposed animals 3 months after exposure. (A) *Gad1* transcript levels were lower in the PB-treated group 3 months after drug exposure ($F_{2,20}$=8.199; P=0.0025; PB v VEH: 0.055, Dunnett's test). This was consistent with the pattern we observed 3 days after drug exposure. (B) *pvalb* was *increased* in the LEV-treated group compared to vehicle or PB exposure ($F_{2,20}$=4.23; P=0.029; PB v LEV: 0.02, Dunnett's test) differing from what we observed 3 days after exposure, and the PB-treated group did not differ from controls (P=0.60), differing from what we observed 3 days after exposure. (C-E) *Gfap, opalin and cd68* did not differ across treatment groups at 3 months post exposure. (*Gfap*: $F_{2,23}$=0.58, p=0.57; *Opalin*: $F_{2,20}$=0.12, p=0.89; *cd68*: $F_{2,20}$=0.049, p=0.95).
